# GARP promotes the proliferation and therapeutic resistance of bone sarcoma cancer cells through the activation of TGF-β

**DOI:** 10.1038/s41419-020-03197-z

**Published:** 2020-11-17

**Authors:** Ana Belén Carrillo-Gálvez, Juan Esteban Quintero, René Rodríguez, Sofía T. Menéndez, M. Victoria González, Verónica Blanco-Lorenzo, Eva Allonca, Virgínea de Araújo Farias, Juan Elías González-Correa, Nadina Erill-Sagalés, Iñigo Martínez-Zubiaurre, Turid Hellevik, Sabina Sánchez-Hernández, Pilar Muñoz, Federico Zurita, Francisco Martín, Juan Carlos Rodríguez-Manzaneque, Per Anderson

**Affiliations:** 1 Centre for Genomics and Oncological Research (GENYO), PTS Granada, Avenida de la Ilustración 114, 18016 Granada, Spain; 2 Instituto de Investigación Sanitaria del Principado de Asturias, Hospital Universitario Central de Asturias, Avenida de Roma, s/n, 33011 Oviedo, Spain; 3 Instituto Universitario de Oncología del Principado de Asturias, 33006, Oviedo, Spain; 4 CIBER en oncología (CIBERONC), 28029, Madrid, Spain; 5 Departamento de Cirugía, Universidad de Oviedo, Oviedo, 33006 Spain; 6 Servicio de Anatomía Patológica, Hospital Universitario Central de Asturias, Oviedo, 33011 Spain; 7 Department of Neurosurgery, Mayo Clinic, Jacksonville, FL 32224 USA; 8 ATRYS, Carrer Provença 392 PB, 08025 Barcelona, Spain; 9 Department of Clinical Medicine, Faculty of Health Sciences, UiT-The Arctic University of Norway, 9037 Tromsø, Norway; 10 University Hospital of Northern Norway, 9038 Tromsø, Norway; 11 Departamento de Genética e Instituto de Biotecnología, Centro de Investigación Biomédica. Universidad de Granada, Granada, Spain; 12 Servicio de Análisis Clínicos e Inmunología, UGC Laboratorio Clínico, Hospital Universitario Virgen de las Nieves, Av. de las Fuerzas Armadas 2, Granada, 18014 Spain; 13 Instituto de Investigación Biosanitaria (ibs.GRANADA), Granada, Spain

**Keywords:** Predictive markers, Prognostic markers, Bone cancer, Sarcoma

## Abstract

Sarcomas are mesenchymal cancers with poor prognosis, representing about 20% of all solid malignancies in children, adolescents, and young adults. Radio- and chemoresistance are common features of sarcomas warranting the search for novel prognostic and predictive markers. GARP/LRRC32 is a TGF-β-activating protein that promotes immune escape and dissemination in various cancers. However, if GARP affects the tumorigenicity and treatment resistance of sarcomas is not known. We show that GARP is expressed by human osteo-, chondro-, and undifferentiated pleomorphic sarcomas and is associated with a significantly worse clinical prognosis. Silencing of GARP in bone sarcoma cell lines blocked their proliferation and induced apoptosis. In contrast, overexpression of GARP promoted their growth in vitro and in vivo and increased their resistance to DNA damage and cell death induced by etoposide, doxorubicin, and irradiation. Our data suggest that GARP could serve as a marker with therapeutic, prognostic, and predictive value in sarcoma. We propose that targeting GARP in bone sarcomas could reduce tumour burden while simultaneously improving the efficacy of chemo- and radiotherapy.

## Introduction

Primary bone sarcomas, which mainly includes osteosarcoma, chondrosarcoma, and Ewing sarcoma, are a group of rare tumours of mesenchymal or ectodermal origin that constitutes <0.2% of all malignancies in adults^1^. However, high grade osteo- and Ewing sarcomas mostly affect children and young adults (<20 years) where they account for about 3.5% and 2%, respectively, of all solid cancers^2^. Although advances in surgery and multi-agent chemotherapy have markedly improved prognosis and overall survival of bone sarcoma, the 5-year survival rate remains at 60–70% for patients with localised disease, and as low as 20–30% for patients with metastatic disease^3–5^. The poor prognosis of bone sarcomas is in part linked to resistance to conventional chemotherapy (CT) and radiation therapy (RT)^6–8^. This makes cancers in the bone and cartilage the third most important cause of cancer-related death in children and adolescents in the United States^9^. Thus, there is an urgent need for new therapies, biomarkers (diagnostic, prognostic, or therapeutic), and a greater understanding of the mechanisms behind the chemo- and radioresistance of bone sarcomas.

Transforming growth factor (TGF)-β is a pleiotropic cytokine that regulates proliferation, angiogenesis, carcinogenesis, and immune responses^10^. TGF-β is produced as an inactive complex consisting of the mature TGF-β homodimer bound to the latency-associated peptide (LAP). A conformational change in the LAP induced by integrins, proteases, or reactive oxygen species releases TGF-β^11^, which then can bind its receptors and induce signalling via canonical (mothers against decapentaplegic homolog; SMADs) and non-canonical pathways^12^. While TGF-β initially acts as a tumour suppressor, at later stages of carcinogenesis it promotes tumour growth and metastasis via induction of epithelial-to-mesenchymal transition (EMT), inhibition of the anti-tumour immune response, and the recruitment/induction of cancer-associated fibroblasts into the tumour microenvironment^13^. Furthermore, recent data suggest that TGF-β also can play a role in the chemo- and radioresistance of tumours^14^.

Glycoprotein A repetitions predominant (GARP)/leucine rich repeat containing 32 (LRRC32)^15^ binds and activates latent TGF-β1 on the surface of regulatory T cells (Tregs) and B cells, promoting their immunosuppressive function and isotype switching, respectively^16–18^. GARP-overexpression in colorectal and lung cancer is associated with a worse prognosis due to a TGF-β-dependent pro-metastatic effect and to the induction of an immunosuppressive tumour microenvironment^19–21^.

Mesenchymal stem cells (MSCs) are non-haematopoietic, multipotent cells that can give rise to bone, cartilage, and adipocytes^22^. MSCs are a key component of the bone marrow (BM) where they regulate haematopoiesis^23^ and participate in bone homoeostasis^13^. Abundant evidence suggests that oncogenic events during the differentiation of MSCs can give rise to sarcomas^24,25^. We recently found that adipose tissue-derived MSCs (ASCs) express GARP in vitro, which binds latent TGF-β1 to their surface^26,27^. However, whether GARP is expressed on bone sarcoma cells and if GARP can promote their tumorigenicity and/or chemo/radioresistance are unknown.

In this work, we show that GARP is expressed on several established and freshly isolated patient-derived bone sarcoma primary cell lines. Silencing of GARP inhibited their proliferation, whereas overexpression of GARP increased their proliferation in vitro and in vivo, via a TGF-β-dependent mechanism. GARP-overexpressing bone sarcoma cells were more resistant to etoposide-, doxorubicin-, and radiation-induced cell death in vitro. Importantly, GARP was found to be overexpressed in human osteo-, chondro-, and pleomorphic sarcomas and was associated with a significantly worse clinical prognosis. Our data suggest that GARP might serve as a target and/or biomarker with therapeutic, prognostic, and predictive value for bone sarcoma tumours.

## Materials and methods

### Cell cultures

The osteosarcoma cell lines G292, T1–73, and SAOS-2 were obtained from the American Type Culture Collection (ATCC). The OST-3 and OST-4 primary cell lines were generated from tumour samples surgically resected at the Hospital Universitario Central de Asturias (Oviedo, Spain). OST-3 derives from a conventional osteoblastic osteosarcoma resected from a 10-year-old female patient and OST-4 corresponds to a dedifferentiated osteosarcoma from a 69-year-old female patient. The chondrosarcoma cell line T-CDS17 was previously described^28^. All cell lines were cultured in Advanced DMEM (Invitrogen) with 10% FBS (Biowest), 1% Glutamax (Gibco, Thermo Fisher Scientific), and 100 U/ml penicillin/streptomycin (Gibco, Thermo Fisher Scientific) at 37 °C, 5% CO_2_. The Ewing’s sarcoma cell line RD-ES was cultured in RPMI-1640 (Gibco, Thermo Fisher Scientific) supplemented with 10% FBS and 100 U/ml penicillin/streptomycin. All cell lines were routinely tested for mycoplasma contamination. The analysis of GARP expression by RT-PCR and flow cytometry, the production of lentiviral vectors (LVs) and transduction of cells, the analysis of cell proliferation in vitro, cell viability in response to etoposide and doxorubicin, the measurement of double-strand DNA breaks (DSBs), and the production of active TGF-β are described in detail in the Supplementary materials and methods.

### Analysis of tumour growth in vivo

Female NOD scid gamma (NSG) mice (8–12 weeks) were obtained from the Centro de Investigaciones Biomédicas (CIBM, Granada, Spain). All experiments were performed according to the Institutional Guidelines for the Care and Use of Laboratory Animals in Research, with the approval of the Ethics Committee in Animal Research at the University of Granada (Granada, Spain). A pilot experiment was performed using NT G292 cells in order to determine the optimal cell number needed to obtain a controlled and reproducible tumour growth over time, and thus sample size according to the replacement, replacement and reduction framework for ethical animal use. Mice were allocated into four groups using complete randomisation. Non-transduced (NT), LV-CTRL (cells transduced with a LV encoding a non-targeting shRNA), GARP^KO2^ (cells transduced with an LV encoding a GARP-specific shRNA), and GARP^++^ (cells transduced with an LV encoding a GARP cDNA) G292 (4 × 10^6^) cells, in PBS, were injected subcutaneously and tumour growth and body weight were measured every 6–7 days in a non-blinded fashion. Tumour volume was calculated using the formula: (π/6) × (*a*^2^ × *b*) where *a* and *b* represent the values for the smaller and the larger tumour diameter, respectively. After 2–3 months (or when the tumour volume reached 1800 mm^3^), mice were sacrificed, tumours were removed and tumour volumes and weights were measured. Pre-established criteria for exclusion included a 15% loss of total body weight, breathing difficulties, persistent lordosis, continuous salivation, or convulsions. Immunohistochemistry was performed on paraffin-embedded tissue sections using monoclonal antibodies against human Ki67 (MIB-1, DAKO/Agilent, Santa Clara, CA, Agilent, Cat#: F726801) and phosphorylated-SMAD3 (phosphoS423 + S425, EP823Y, Abcam, Cambridge, UK, Abcam, Cat#: 1880-1) as described in Supplementary materials and methods.

### Clonogenic assay

Non-transduced (NT) and GARP-overexpressing (GARP^++^) SAOS-2 and RD-ES cells were added to 6-well plates at various densities: 2000, 4000, 8000, and 160,000 cells/well (NT) and 1000, 2000, 4000, 8000 cells/well (GARP^++^). Cells were exposed to γ-radiation using a L. Shepherd & associates MARK-I model 30 Caesium-137 irradiator at the Experimental Radiology Unit, University of Granada (Spain), with single fractions of 0, 2, 4, and 8 Gy, using a dose rate of 1.66 Gy min^-1^. In some experiments, SB431542 (10 µM) was added 24 h before irradiation. Cells were maintained in culture until the appearance of countable colonies (7–9 days following irradiation). Cells were fixed and stained with crystal violet and colonies counted (colonies with >50 cells/colony were scored for survival). The surviving fraction was calculated as previously described^29^.

### Patients, tissue specimens, and IHC

Paraffin-embedded tissues from 89 patients with sarcoma who underwent resection of their tumours at the Hospital Universitario Central de Asturias (HUCA) were studied. Samples and clinical data from donors included in this study were provided by the Principado de Asturias BioBank (PT17/0015/0023) integrated in the Spanish National Biobanks Network and they were processed following standard operating procedures with the appropriate approval of the Ethical and Scientific Committees. All samples from human origin were obtained upon signed informed consent. Sixty percent of the cases were men; mean age at diagnosis was 49 years (range 2–89 years). Twenty eight (31%) patients had a history of tobacco consumption (15 current and 13 former smokers). Tumour grade was evaluated in H&E-stained preparations using the French Federation of Comprehensive Cancer Centres grading system^30^. Clinicopathological features of the patients are included in Table S1. Construction of the tissue microarray (TMA) and the staining of the TMA for GARP and subsequent scoring are described in Supplementary materials and methods.

### Statistical analysis

For the in vitro experiments and the tumour growth experiment in vivo, the statistical analysis was performed using the GraphPad Prism software (GraphPad Software, Inc, La Jolla, CA). All data are represented as mean (SD) of at least three independent experiments unless otherwise stated in the figure legend. Data sets were tested for normality using the Shapiro-Wilk test. A Student’s *t*-test was used for pairwise comparisons while multiple comparisons of the data were performed using the one-way ANOVA, followed by the Bonferroni post test. According to both tests there were no significant differences in variance between the compared groups. The statistical analysis of the clinical data was carried out using the software package SPSS 24 (SPSS, IBM corp.). The experimental results distributed among the different clinical groups of tumours were tested for significance employing the *χ*^2^-test (with Yates’ correction, when appropriate). For statistical purposes continuous variables (age, tumour size) were dichotomised, taking the median value as a cut-off point. Survival curves were calculated using the Kaplan-Meier product limit estimate. Differences between survival times were analysed by the log-rank method and the hazard ratio was calculated by univariate Cox regression analysis. Multivariate Cox proportional hazards models (forward Wald method) were used to examine the relative impact of those variables statistically significant in univariate analysis. All tests were two-sided and *P* values < 0.05 were considered statistically significant.

## Results

### GARP is expressed on several bone sarcoma cancer cell lines and its silencing blocks their proliferation

**Fig. 1:**
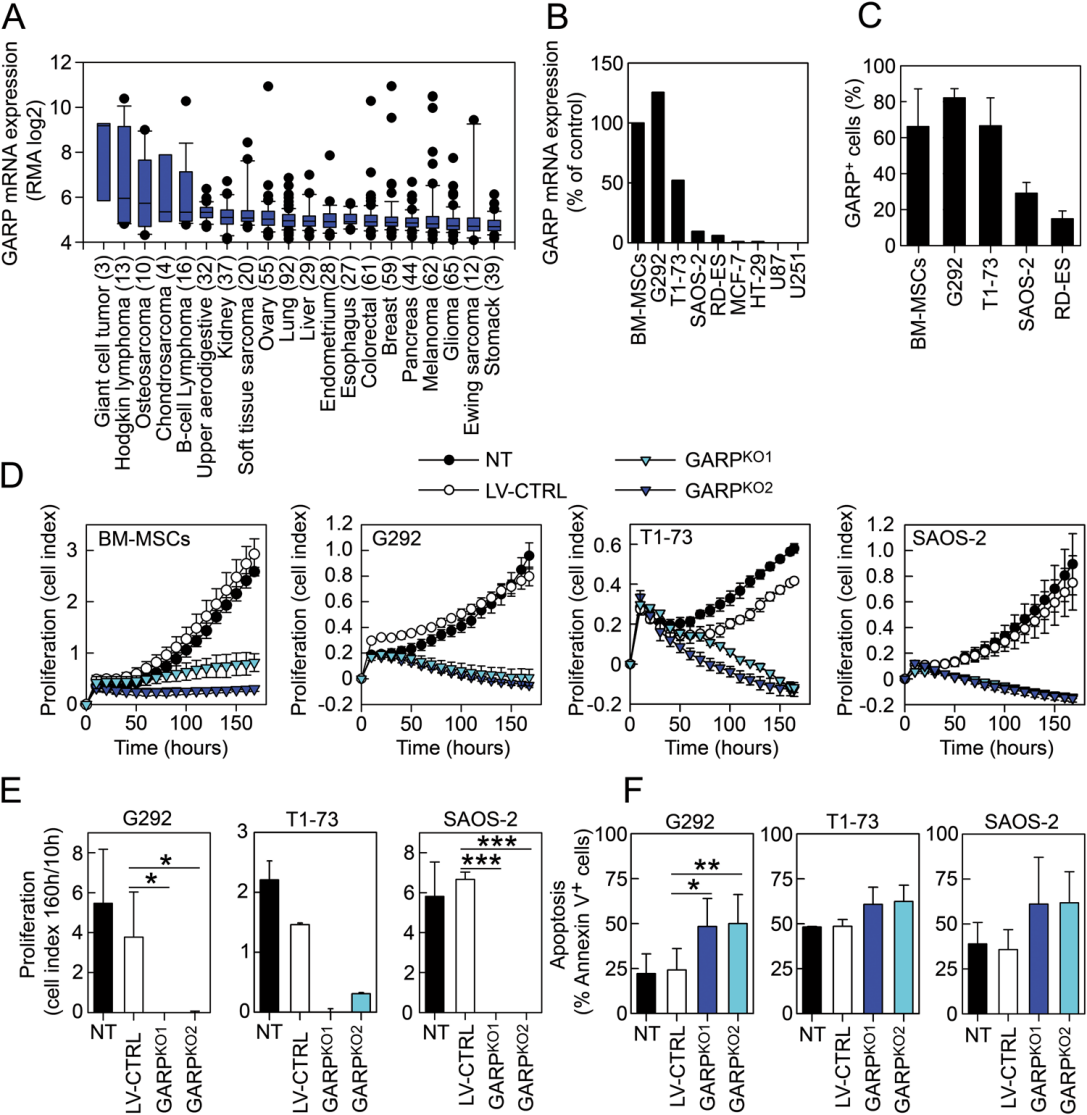
GARP is expressed on several bone sarcoma cancer cell lines and its silencing blocks their proliferation. **A** GARP mRNA expression data from various cancer cell lines retrieved from the CCLE data base. The numbers in parentheses indicate the number of cell lines analysed/cancer type. RMA log2 mRNA expression values are shown as box plots and sorted by the median in descending order from left to right. Outliers are plotted as filled circles. **B** GARP mRNA expression in primary BM-MSCs and various cancer cell lines. Data are shown as mean (SD), relative to the GARP expression in BM-MSCs. **C** Summary of GARP expression (% in relation to isotype control) on primary BM-MSCs and selected bone sarcoma cell lines. Data are shown as mean (SD) of three individual stainings. **D** Silencing of GARP in BM-MSCs and the osteosarcoma cell lines G292, T1–73, and SAOS-2 inhibits their proliferation. Proliferation (cell index) of non-transduced (NT), LV-CTRL, GARP^KO1^, and GARP^KO2^ cells was measured in real-time during 7 days. Data are shown as mean (SD) of experimental duplicates. One representative experiment out of three is shown. **E** Graphs show the mean (SD) of the proliferation represented as cell index at 160 h divided by the corresponding cell index at 10 h of three individual experiments. * =*P* < 0.05; *** =*P* < 0.001. **F** Silencing of GARP in osteosarcoma cell lines induce cell death by apoptosis. Apoptosis was measured in NT, LV-CTRL, GARP^KO1^, and GARP^KO2^ G292 (left graph), T1–73 (middle graph), and SAOS-2 (right graph) cells by staining the cells with 7AAD/Annexin V and analysing the cells by flow cytometry. Data are shown as mean (SD) of three independent experiments. * =*P* < 0.05.

An analysis of the Cancer Cell Line Encyclopedia (CCLE) database revealed that elevated GARP mRNA expression could be found in several sarcoma subtypes, including giant cell tumours, osteosarcomas, and chondrosarcomas, with relatively lower levels in carcinoma and glioma cell lines (Fig. 1A). These data were corroborated by a GARP qPCR on human bone marrow-derived (BM)-MSCs, osteosarcoma cells (G292, T1–73, and SAOS-2), an Ewing sarcoma cell line (RD-ES), two glioblastoma cell lines (U87, U251), and two carcinoma cell lines (HT-29, MCF-7) (Fig. 1B) and by flow cytometry (Figs. 1C and S1A). Silencing of GARP in BM-MSCs, G292, T1–73, and SAOS-2 cells (Figs. S1B, S1C, and S2), using LVs encoding for two distinct GARP-specific shRNAs (GARP^KO1^ and GARP^KO2^), decreased their proliferative capacity compared to non-transduced (NT) and control transduced (LV-CTRL) cells (Fig. 1D, E) and increased cell death by apoptosis (Fig. 1F).

### GARP promotes the proliferation of bone sarcoma cell lines through activation of TGF-β

**Fig. 2:**
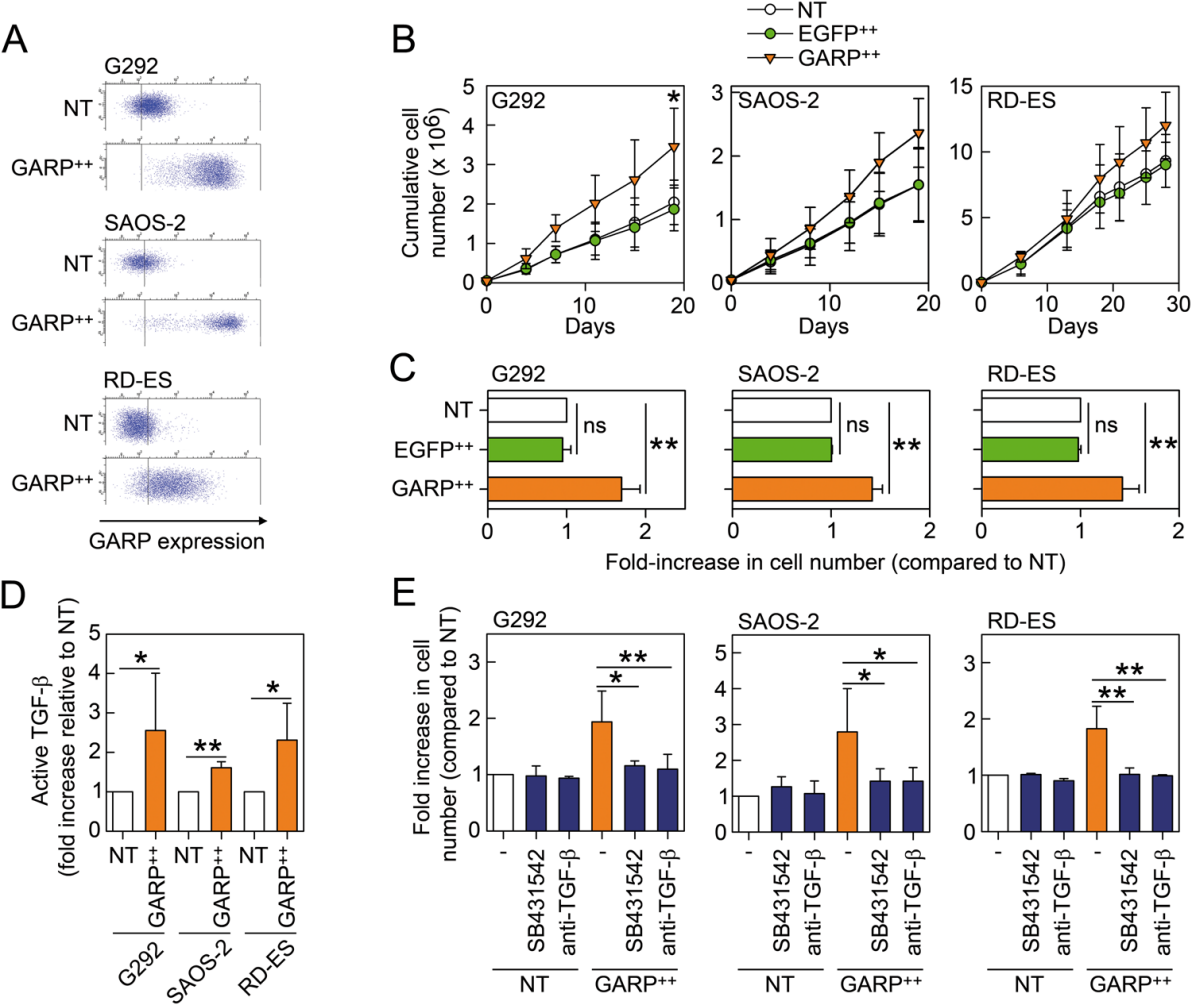
GARP promotes the proliferation of bone sarcoma cell lines through the activation of TGF-β. **A** Overexpression of GARP in bone sarcoma cell lines. Representative dot plots of non-transduced (NT) and GARP-overexpressing (GARP^++^) G292 (top panel), SAOS-2 (middle panel), and RD-ES (bottom panels). Corresponding isotype staining is represented by vertical lines in all plots. **B** GARP-overexpression increases the proliferation of bone sarcoma cell lines. Growth curves of NT, GARP^++^, and EGFP-overexpressing (EGFP^++^) G292, SAOS-2, and RD-ES cells were generated by plotting the cumulative cell numbers vs. time. Data are shown as mean (SD) of three individual experiments. * =*P* < 0.05. **C** The fold-increase in cell numbers of EGFP^++^ and GARP^++^ cells in relation to NT cells at the end of each experiment are shown as mean (SD) of three individual experiments. ** =*P* < 0.01. **D** GARP^++^ sarcoma cells produce more active TGF-β compared to NT cells. NT and GARP^++^ G292, SAOS-2, and RD-ES cells were co-cultured for 24 h with SBE-HEK293 cells subsequently assayed for luciferase activity. The fold-increase in TGF-β activity of the GARP^++^ cells is relative to the NT for each cell line. Data are represented as mean (SD) of three independent experiments. * =*P* < 0.05, ** =*P* < 0.01. **E** The increased proliferation of GARP^++^ sarcoma cells depends on TGF-β. NT and GARP^++^ G292, SAOS-2, and RD-ES were cultured in the absence or presence of SB431542 or anti-TGF-β1/2/3 Ab (11D1) for 3 days and subsequently counted. Fold-increase in cell number is relative to NT for each cell line. Data are shown as mean (SD) of three independent experiments. * =*P* < 0.05; ** =*P* < 0.01.

We next analysed the effect of GARP-overexpression on the proliferation of various bone sarcoma cells. To this end, we overexpressed GARP (GARP^++^) in G292, SAOS-2, and RD-ES cells using a LV encoding a codon-optimised human GARP cDNA under the control of the CMV promoter (Fig. 2A). Parallelly, control cells were transduced with LV encoding for the enhanced green fluorescent protein (EGFP^++^). GARP^++^ cells exhibited an increased proliferative capacity compared to NT and EGFP^++^ cells (Fig. 2B, C). We next assessed whether GARP-overexpression could increase the activation of TGF-β. No active TGF-β was detected in non-acidified supernatants from NT and GARP^++^ sarcoma cells by conventional ELISA (data not shown). However, using a TGF-β activity reporter cell line, we found that GARP^++^ cells produced significantly higher levels of active TGF-β compared to NT cells (Figs. 2D and S3). Blocking TGF-β signalling with SB431542 or neutralising TGF-β using an anti-TGF-β1/2/3 antibody significantly reduced the proliferation of the GARP^++^ cells (Fig. 2E).

### GARP increases the growth of G292 tumours in vivo

**Fig. 3:**
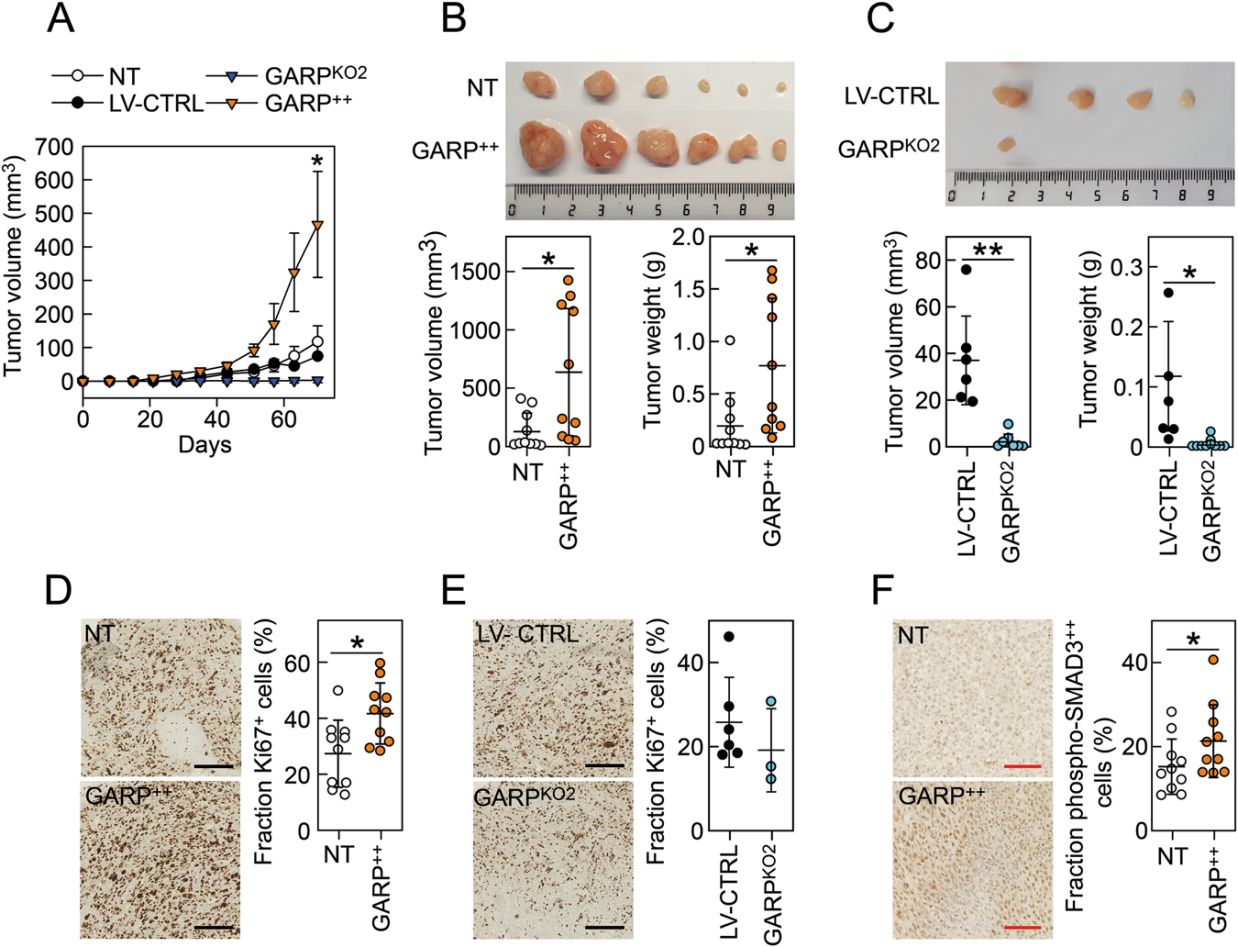
GARP increases the growth of G292 tumours in vivo. **A** NT (*N* = 10), LV-CTRL (*N* = 6), GARP^KO2^ (*N* = 10), and GARP^++^ (*N* = 10) G292 cells were injected subcutaneously into NSG mice and tumour growth was followed during 70 days. The tumour height and width were measured weekly using an automatic calliper and tumour volumes were calculated as described in materials and methods. Data are shown as mean (SD). * =*P* < 0.05 GARP^++^ vs. NT. **B** Representative image of isolated NT and GARP^++^ tumours at the end of the experiment (top panel). The volumes (left graph) and weights (right graph) of the isolated tumours were measured as described in materials and methods. Data are shown as mean (SD) with white (NT) and orange (GARP^++^) circles representing individual tumours. **C** Representative image of isolated LV-CTRL and GARP^KO2^ tumours at the end of the experiment (top panel). The volumes (left graph) and weights (right graph) of the isolated tumours were measured as described in materials and methods. Data are shown as mean (SD) with black (LV-GARP) and blue (GARP^KO2^) circles representing individual tumours. **D** The proliferation of tumour cells were analysed by an IHC staining of Ki67 in NT (*N* = 10) and GARP^++^ (*N* = 10) tumours. Data are shown as mean (SD) with white (NT) and orange (GARP^++^) circles representing individual tumours. * =*P* < 0.05. **E** Staining of Ki67 in LV-CTRL (*N* = 6) and GARP^KO2^ (*N* = 3) tumours. Data are shown as mean (SD) with black (LV-CTRL) and blue (GARP^KO2^) circles representing individual tumours. **F** Activation of the TGF-β signalling pathway was analysed by phospho-SMAD3 IHC in NT and GARP^++^ G292 tumours. Data are shown as mean (SD) with open (NT) and orange (GARP^++^) circles representing individual tumours. * =*P* < 0.05. Scale bars: 250 μm (black) or 100 μm (red).

In order to analyse whether the expression levels of GARP are also important for tumour growth in vivo, we injected NT, GARP^++^, LV-CTRL, and GARP^KO2^ G292 cells subcutaneously into NSG mice and followed tumour growth over time. GARP^++^ G292 tumours exhibited a significantly increased growth in vivo compared to NT G292 tumours, whereas smaller tumours were found in only three out of eight mice injected with GARP^KO2^ G292 cells (Fig. 3A–C). Furthermore, both volume and weight of GARP^++^ tumours isolated from the sacrificed mice were significantly increased compared to NT tumours (Fig. 3B), whereas GARP^KO2^ tumours were significantly smaller compared to LV-CTRL tumours (Fig. 3C). GARP^++^ tumours contained significantly higher levels of proliferating Ki67^+^ cells compared to NT tumours while an opposite trend was observed between GARP^KO2^ and LV-CTRL tumours. Finally, we analysed the activation of SMAD3 in the GARP^++^ and NT tumours by IHC and observed an increased fraction of cells with high SMAD3-phosphorylation in GARP^++^ tumours compared to NT tumours (Fig. 3F).

### GARP-overexpressing bone sarcoma cell lines are more resistant to etoposide- and doxorubicin-induced cell death: dependence on TGF-β

**Fig. 4:**
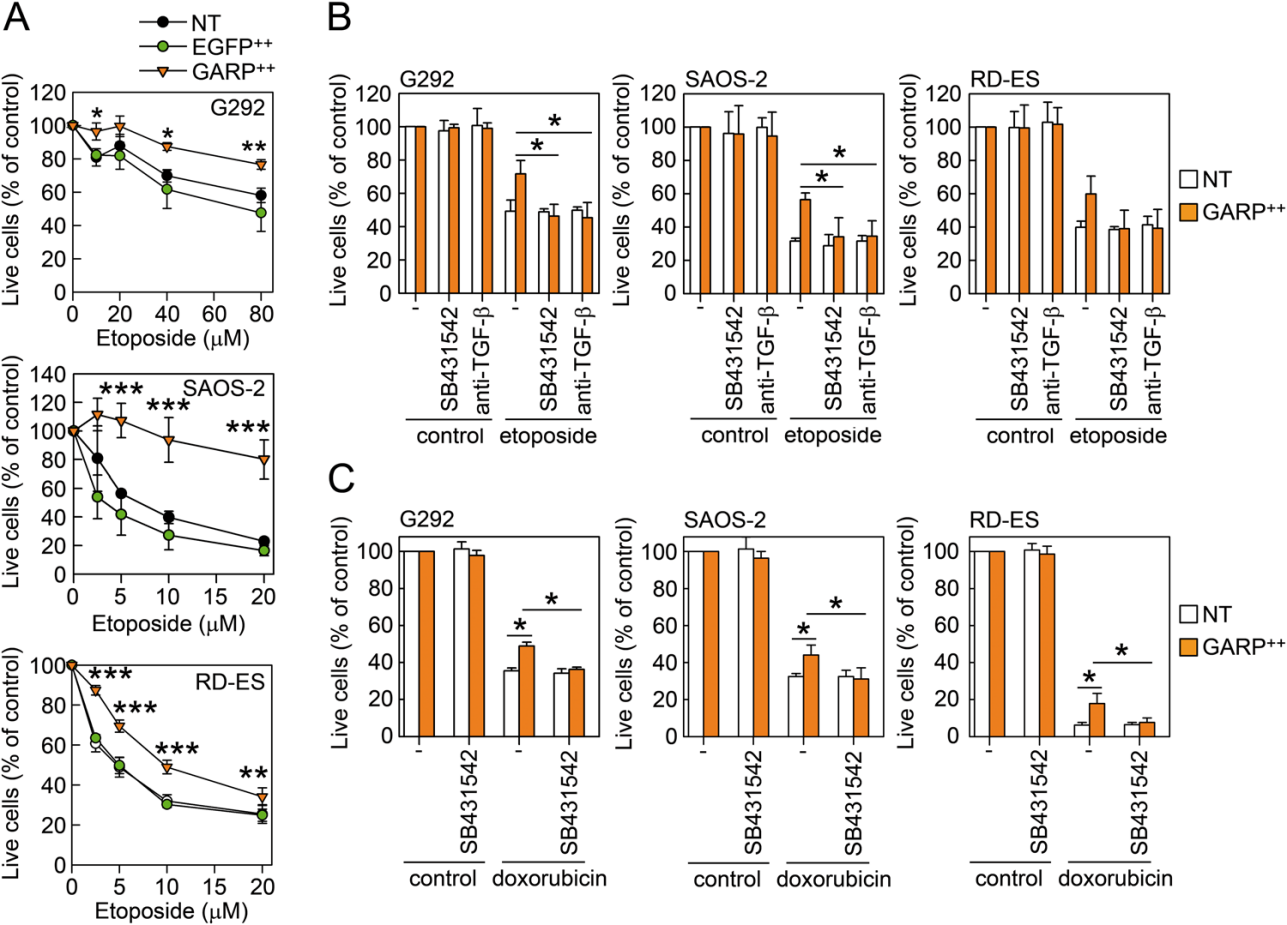
GARP-overexpressing bone sarcoma cell lines are more resistant to etoposide- and doxorubicin-induced cell death. Non-transduced (NT), GARP-overexpressing (GARP^++^), EGFP-overexpressing (EGFP^++^) G292, SAOS-2, and RD-ES were subjected to different concentrations of etoposide in vitro. **A** Cell viability was measure using CelltiterBlue and plotted as % of control (cells without etoposide). Data are shown as mean (SD) of three individual experiments. * =*P* < 0.05; ** =*P* < 0.01; *** =*P* < 0.001. **B** NT and GARP^++^ G292, SAOS-2, and RD-ES cells were subjected to 80 µM (G292) or 10 µM (SAOS-2 and RD-ES) etoposide, with or without SB431542 and anti-TGF-β1/2/3 Ab (11D1). Cell viability was analysed using CelltiterBlue and plotted as % of control (cells without etoposide). Data are shown as mean (SD) of three individual experiments. * =*P* < 0.05. **C** NT and GARP^++^ G292, SAOS-2, and RD-ES cells were subjected to 2.5 µM doxorubicin, with or without SB431542. Cell viability was analysed using CelltiterBlue and plotted as % of control (cells without doxorubicin). Data are shown as mean (SD) of three independent experiments. * =*P* < 0.05.

Previous studies on GARP in the context of cancer have focused on the inhibition of the immune response through increased activation of TGF-β, especially the induction of Foxp3^+^ Tregs^19–21^. However, to our knowledge, no study has analysed the effect of GARP expression on the resistance to chemotherapy or radiation. To this end, we added different concentrations of the DNA damaging agent etoposide to NT, EGFP^++^, GARP^++^ G292, SAOS-2, and RD-ES cells and analysed cell survival and apoptosis. We found that GARP-overexpression increased cell survival (Fig. 4A) and reduced apoptosis (Fig. S4) of all the tumour cell lines. Inhibition of TGF-β signalling (SB431542) or neutralisation of TGF-β (1D11) significantly reversed the survival of GARP-overexpressing cells, suggesting a mechanism dependent on TGF-β (Fig. 4B). Similarly, GARP^++^ cells were more resistant to cell death induced by doxorubicin, although to a lesser extent compared to etoposide. Again, inhibition of TGF-β signalling reversed the protective effect conferred by GARP (Fig. 4C).

### GARP-overexpressing sarcoma cells display reduced sensitivity to radiation-induced cell death and DNA damage

**Fig. 5:**
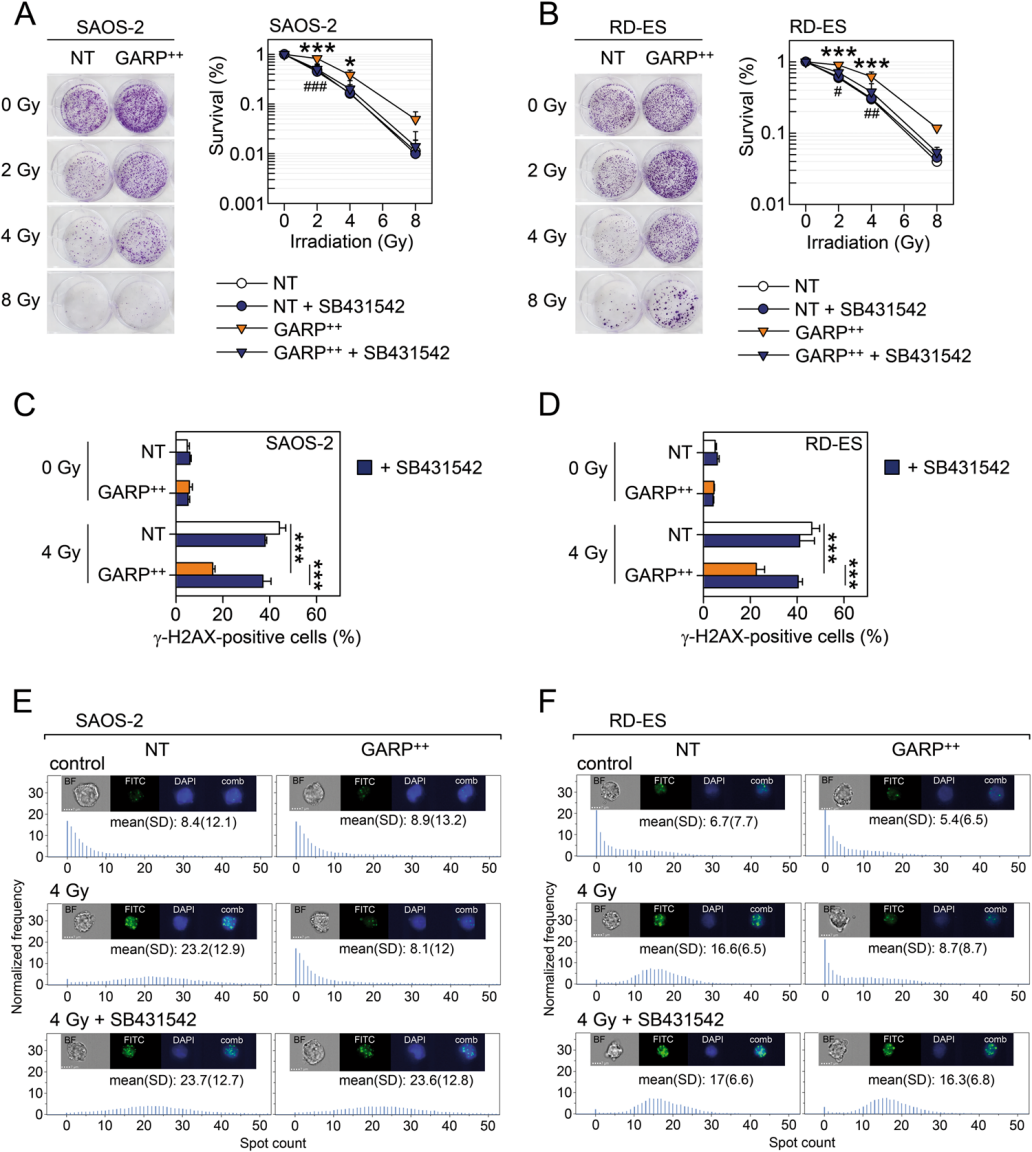
GARP-overexpressing bone sarcoma cell lines are more resistant to radiation-induced cell death and DNA damage. The susceptibility to irradiation was analysed using a clonogenic assay. NT and GARP^++^ SAOS-2 (**A**) and RD-ES cells (**B**) were seeded at low density in 6-well plates and cultured with or without SB431542 as described in materials and methods. The cells were irradiated with 2, 4, and 8 Gy. After 2 weeks, colonies were fixed, stained with crystal violet and counted as described in materials and methods. Data are plotted as survival fraction (relative to non-irradiated cells) and shown as mean (SD) of three independent experiments. * =*P* < 0.05; *** =*P* < 0.001 GARP^++^ vs. NT, # =*P* < 0.05; ## =*P* < 0.01; ### =*P* < 0.001 GARP^++^ + SB431542 vs. GARP^++^. NT and GARP^++^ SAOS-2 (**C**) and RD-ES (**D**) cells were cultured with or without SB431542 and irradiated with 4 Gy and analysed for γ-H2AX by flow cytometry. Data are shown as mean (SD) of three independent experiments. NT and GARP^++^ SAOS-2 (**E**) and RD-ES (**F**) cells were cultured with or without SB431542 and irradiated with 4 Gy and analysed for γ-H2AX using an imagestream flow cytometer. The number of γ-H2AX foci/nucleus is shown as mean (SD). One representative experiment out of two is shown.

We next performed a clonogenic assay to assess the relative resistance of NT and GARP^++^ sarcoma cells to radiation-induced cell death. G292 cells did not form countable colonies, therefore only the SAOS-2 and RD-ES cell lines were used. The NT and GARP^++^ cell lines were subjected to different single doses of radiation (2, 4, and 8 Gy), in the presence or absence of SB431542. Colony counting showed that GARP^++^ cells were more resistant to radiation-induced cell death compared to NT cells. Inhibition of TGF-β signalling abrogated the GARP-mediated radioresistance in both cell lines (Fig. 5A, B).

TGF-β has been shown to protect cells against radiation-induced DNA damage through its effect on DNA repair^31,32^. We thus exposed NT and GARP^++^ SAOS-2 and RD-ES cells, cultured with or without SB431542 for 24 h, with a single dose of radiation (4 Gy). We then analysed the induction of DNA damage, as visualised by the phosphorylation of the H2A histone family member X (γ-H2AX), before and 2 hours after radiation using a conventional flow cytometer (Fig. 5C, D) or an Imagestream flow cytometer in order to visualise the nuclear foci of γ-H2AX (Fig. 5E, F). We found that GARP^++^ cells were more resistant to radiation-induced DNA damage in a TGF-β-dependent manner (Fig. 5C–F).

### GARP is highly expressed in human osteo-, chondro-, and pleiomorphic sarcomas and is associated with a poor prognosis

**Fig. 6:**
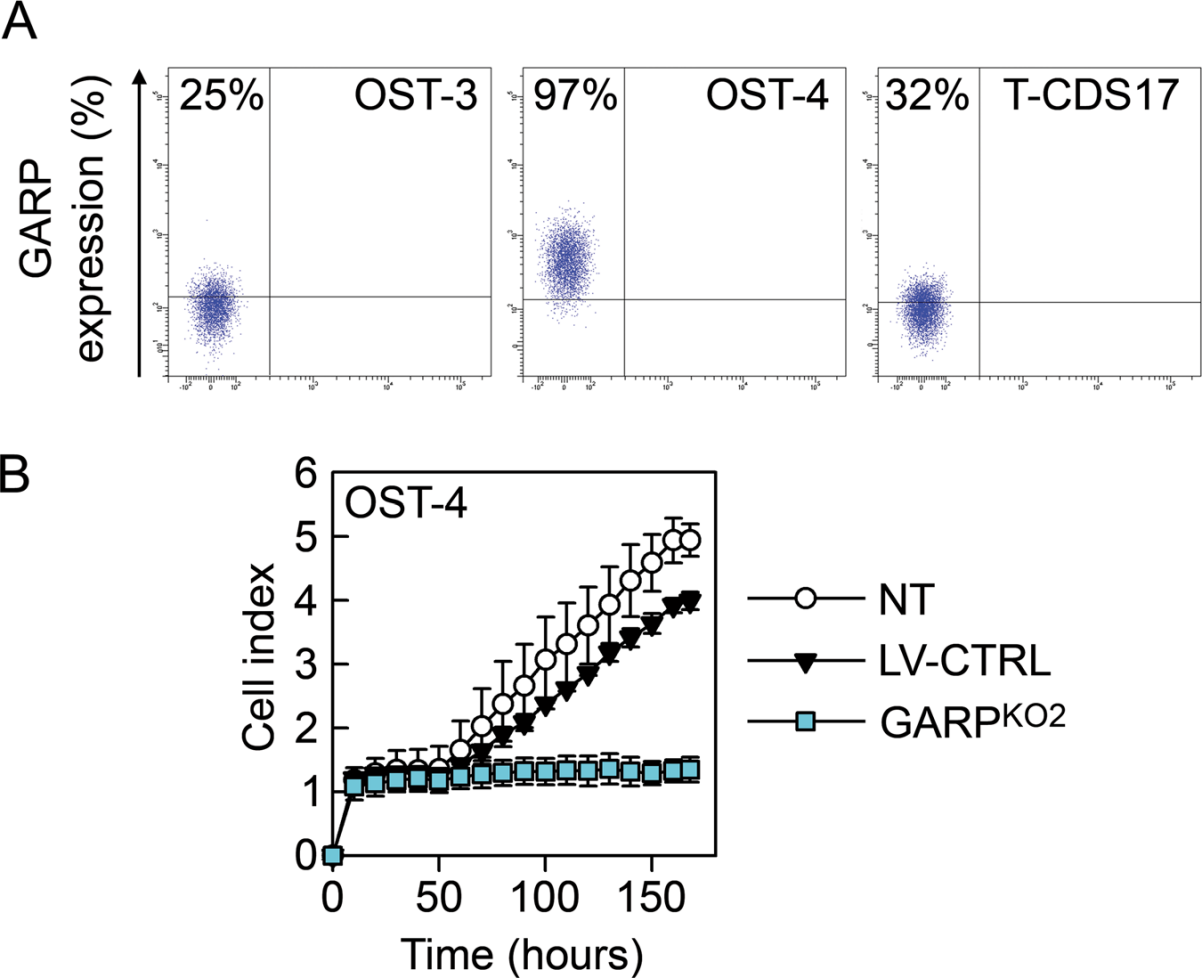
Patient-derived cell lines from osteosarcoma and chondrosarcoma tumours express GARP. **A** Patient-derived tumour cells were derived from two osteosarcoma tumours (OST-3 and OST-4) and one chondrosarcoma tumour (T-CDS17). Cell lines were stained for surface expression of GARP and analysed by flow cytometry. Representative staining are shown. **B** The proliferation of NT, LV-CTRL, and GARP^KO2^ OST-4 cells analysed using xCELLigence real-time cell analyser system. Data are shown as mean (SD) of experimental duplicates. One representative experiment out of two is shown.

The clinical relevance of GARP expression in sarcomas is unknown. Therefore, we studied the expression of GARP in primary human bone sarcomas and its correlation with available clinical data. We first analysed GARP expression on the cell surface on primary patient-derived cell lines from two osteosarcomas (OST-3, OST-4) and one chondrosarcoma (T-CDS17)^28^ by flow cytometry. All three cell lines expressed GARP, albeit at varying levels (Fig. 6A). The OST-4 cell line expressed GARP at a high level and silencing of GARP reduced its proliferation (Figs. 6B and S5A).

**Fig. 7:**
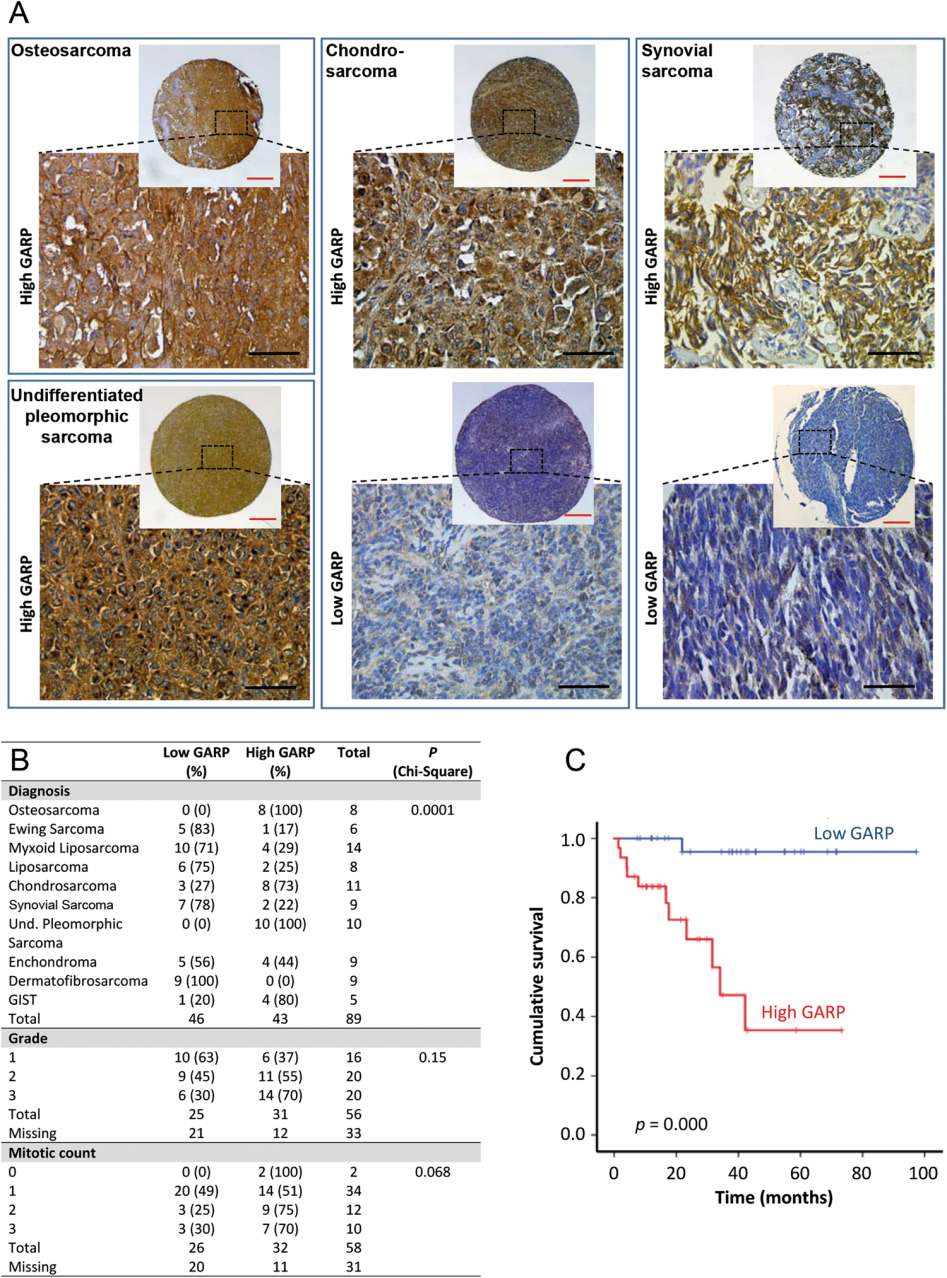
Immunohistochemical analysis of GARP expression in sarcoma tissue specimens and impact on patient survival. **A** Representative examples of the indicated types of sarcoma showing high and low GARP staining. Scale bars: 250 μm (red) or 50 μm (black). **B** Distribution of sarcoma cases (*N* = 89) according to their GARP expression level across categories of the indicated patient characteristics and tumour clinicopathological parameters (a more comprehensive description is presented in Supplementary Tables S1 and S2). *P* values are shown. **C** Kaplan-Meier cumulative survival curves categorised by GARP protein expression in the cohort of sarcoma patients. *P*-values were estimated using the log-rank test.

We next analysed the expression of GARP by immunohistochemistry (IHC) in a collection of human TMAs, including samples from 89 patients of 10 types of sarcomas. GARP was highly expressed in 48% (43/89) of the sarcoma samples, including all osteosarcoma and undifferentiated pleomorphic sarcoma cases (Fig. 7A, B). While 73% (8/11) of chondrosarcomas were positive for GARP, the majority of synovial sarcomas (7/9) and Ewing Sarcomas (5/6) expressed low GARP levels (Fig. 7A, B). None of clinicopathological variables analysed showed a significant correlation with the level of GARP expression (Table S1), although a tendency was observed for a higher grade (*P* = 0.15) and mitotic count scoring (*P* = 0.068) in tumours showing high expression of GARP (Fig. 7B). Interestingly, patient cases expressing low GARP levels had a significantly longer survival time than those with high GARP expression (94 months (CI 87–100) vs. 41 months (CI 28–54), respectively; HR 19; *P* = 0.0001). The 5-year survival rate was 95.5% for low expressing cases and 35.3% for high expressing cases (Fig. 7C). Tumour grade, tumour type, mitotic count, necrosis, and GARP expression level were included in the multivariate survival analysis. Tumour necrosis showed an independent prognostic value (*P* = 0.024, HR 2.643). Importantly, a tendency was observed towards high GARP expression having an independent prognostic value (*P* = 0.056) (Table S2). Finally, in a series of 11 sarcoma patients for which we have response data to different first-line CT treatments, we found that the only complete responses occurred in two of the four patients with low levels of GARP. On the other hand, the only case where the treatment had no effect on tumour growth corresponded to a patient with high levels of GARP. Despite the small number of patients included in this analysis, we found significant differences between patients with high and low levels of GARP when performing a dichotomous analysis of complete responses versus the other response options (partial response, stable disease, and progressive disease) (Tables S3 and S4).

## Discussion

GARP is emerging as a compelling target in cancer, both for its role in the function of Tregs which can suppress anti-tumour immune responses^33^, and for its expression in various cancers^19–21^. Metelli et al. showed that overexpression of GARP in the 4T1 murine mammary carcinoma cell line increased its TGF-β activation, tumour growth, metastasis, and immunoevasive properties^20^. Similarly, Hahn et al. showed that GARP is expressed on human melanoma cells, playing an important role in establishing an immunosuppressive tumour microenvironment^19^. We and others have previously shown that GARP is expressed on human MSCs, with implications for their proliferation, differentiation, and immunomodulation^26,34^. However, whether GARP is expressed by mesenchymal neoplasms and its effect on chemo/radiation-induced cell death are unknown.

First, we found that GARP is expressed on several bone sarcoma cell lines, including primary cell lines from osteo- and chondrosarcoma patients. Silencing of GARP in these cell lines decreased their proliferation, suggesting that GARP could serve as a therapeutic target to inhibit bone sarcoma growth. This is in agreement with Zhou et al. who demonstrated that GARP^low^HeLa cells exhibited a decreased proliferative rate and an accumulation of cell cycle-inhibiting proteins, including TP53, cyclin-dependent kinase inhibitor (CDKN)1 A, and CDKN1B compared to GARP^intermediate^ cells and GARP^high^ cells^35^. Although beyond the scope of the current study, understanding how GARP regulates the proliferation and survival of sarcoma cells warrants further investigation.

Second, we show that GARP promotes the capacity of sarcoma cells to activate TGF-β which suggests that G292, SAOS-2, and RD-ES cells express the molecules necessary for GARP-mediated activation of TGF-β^36^. Furthermore, we demonstrate that GARP-overexpressing cells exhibit an increased proliferative capacity, which depended on TGF-β. Interestingly, blocking TGF-β signalling in NT sarcoma cells did not affect their proliferation, despite their basal production of active TGF-β. This could be due to shortcomings of the experimental setup and to the fact that GARP-mediated activation of TGF-β occurs in a cell-contact-dependent manner, inducing a response that differs from that of TGF-β activated in the absence of GARP^13,36^.

Third, we found that high GARP expression on human sarcoma tumours correlated with a significantly worse overall survival. Although none of the clinicopathological variables analysed in this study showed a significant correlation with the level of GARP expression (Table S1), a tendency was nevertheless observed for a higher grade (*P* = 0.15) and mitotic count scoring (*P* = 0.068). These data are in agreement with the pro-proliferative properties of GARP seen in our in vitro and in vivo experiments. Importantly, a tendency was observed towards GARP expression having an independent prognostic value (*P* = 0.056). However, to establish GARP as a prognostic factor for sarcomas, further correlations between GARP expression and outcome need to be performed on additional sarcoma samples.

The current treatment of bone sarcoma is surgery and multimodal CT, and in some cases RT. In general, doxorubicin, cisplatin, and iphosphamide represent first-line drugs for the treatment of localised bone sarcoma while cyclophosphamide in combination with etoposide are important for the treatment of recurrent osteosarcoma and Ewing sarcoma^37^. As for most solid tumours, the combination of improved surgical procedures with efficient CT and high-precision RT has substantially increased the survival rate of bone sarcoma patients. However, during the last 30 years little progress has been achieved and patients with metastatic or recurrent diseases still have a <20% chance of long-term survival despite aggressive therapies^37^. We show that GARP-overexpressing SAOS-2 and RD-ES cells, via TGF-β activation, are more resistant to DNA damage and cell death induced by irradiation and etoposide/doxorubicin in vitro. In addition, in a small group of sarcoma patients we observed a correlation between GARP expression levels and the response to CT. However, due to the small sample size, variety of sarcoma subtypes, and CT modalities, future studies need to analyse the clinical response of GARP^high^ and GARP^low^ sarcomas to CT and RT, using larger and more homogenous patient cohorts.

Due to the crucial involvement of TGF-β in cancer progression and metastasis, there is a great interest in developing enhanced anti-cancer therapies based on the inhibition of TGF-β production or signalling (reviewed in^38^). However, current anti-TGF-β antagonists do not distinguish between homeostatic and disease-induced TGF-β activity and carry the risk of inducing inflammation, autoimmunity, or cardiovascular defects. Several clinical trials have employed TGF-β inhibitors for the treatment of cancer, with positive results achieved for trabedersen and galunisertib^39^. However, other trials have yielded negative results and the risk of adverse effects remains^40–42^. Although GARP is not ubiquitous as TGF-β, its expression is not exclusive to tumour cells. Thus, the sensitivity of sarcoma cells to GARP inhibition relative to healthy tissue needs to be addressed. In conclusion, we propose that GARP might serve as a prognostic and predictive marker in sarcoma management. We propose that targeting GARP in sarcomas has the potential of decreasing tumour burden and increasing the efficacy of CT and RT. Whether our data on GARP-mediated CT/RT resistance can be extrapolated to other GARP-expressing cancers, including colorectal and lung cancer would be interesting to analyse.

## Supplementary information

Supplementary Materials and Methods

Supplementary Figure Legends

Figure S1

Figure S2

Figure S3

Figure S4

Figure S5

Supplementary Table Legends

Supplementary Table 1

Supplementary Table 2

Supplementary Table 3

Supplementary Table 4
