# Supplementary material for: GARP promotes the proliferation and therapeutic resistance of bone sarcoma cancer cells through the activation of TGF-β
Source: Cell Death Dis. 2020 Nov 17;11(11):985. doi: 10.1038/s41419-020-03197-z (PMC7673987; doi:10.1038/s41419-020-03197-z)
Supplement: Supplementary file 1 — Supplementary Materials and Methods [file 41419_2020_3197_MOESM1_ESM.docx]

**SUPPLEMENTARY MATERIALS AND METHODS**

*Production of lentiviral vectors (LVs) and transduction of cells*

LVs encoding human GARP-specific shRNAs (MISSION® shRNA plasmid DNAs, RefSeq: SHCLND-NM_005512; Sigma-Aldrich, MO, USA) were used, referred to in the manuscript as GARP^KO1^ (TRCN0000005218) and GARP^KO2^ (TRCN0000005219). A MISSION® pLKO.1-puro non-mammalian shRNA control plasmid (Sigma-Aldrich) was used as control, referred in the text as LV-CTRL. A LV encoding the human codon-optimized GARP cDNA, LV-GARP (28) were used to overexpress GARP and the LV-CEWP was used as control. LVs were produced by co-transfecting 293T cells with: 1) LV-CTRL/GARP^KO1^/GARP^KO2^/LV-GARP/LV-CEWP plasmid, 2) packaging plasmid pCMVΔR8.91 and 3) envelope plasmid pMD.G. The LVs were subsequently concentrated (x30) using centrifugal filter devices (Amicon Ultra-15, 100 kD, Merck). GARP was silenced in BM-MSCs, G292, T1-73, SAOS-2 and OST-4 cells as previously described (1). To overexpress GARP (GARP^++^), RD-ES and SAOS-2 cells were transduced once with concentrated (30x) LV-GARP, for 5 hours and subsequently expanded, while G292 cells were transduced twice on consecutive days with LV-GARP (x30). Cells were transduced with LV-CEWP as control. GARP expression was analyzed by flow cytometry 4 days after transduction as described below.

*Detection of surface GARP and sorting of GARP^++^ G292 cells*

Cells were stained with an anti-human GARP-eFluor660 Ab (eBioscience, San Diego, CA, USA, Cat#: 50-9882-42). A rat IgG2a kappa-eFluor660 isotype control (eBioscience, Cat#: 50-4321-82) was used in order to determine the background staining and dead cells were excluded using 7AAD (eBioscience). Cells were acquired on a FACS Canto II cytometer and analyzed using the FACS Diva Software (BD Bioscience, San Diego, CA). After GARP staining of LV-GARP transduced G292 cells, the GARP-overexpressing (GARP^++^) population was isolated using a FACS Aria flow cytometer.

*Quantitative PCR (qPCR)*

Total RNA was isolated using the trizol reagent (Invitrogen) according to the manufacturer´s instructions. RNA samples were reverse-transcribed using the Superscript First-Strand kit (Invitrogen) and qPCRs were performed using the QuantiTect SYBRGreen PCR kit (Qiagen, Valencia, CA) on a Stratagene MX3005P system. The following primers were used: GARP FW: 5´-ACAACACCAAGACAAAGTGC-3´, GARP RV: 5´-ACGAAGTGCTGTGTAGAAGC-3´, GAPDH FW: 5´-ATGGGGAAGGTGAAGGTCG-3´, GAPDH RV: 5´-GGGGTCATTGATGGCAACAATA-3´.

*Measurement of active TGF-β*

NT and GARP^++^ G292, SAOS-2, and RD-ES cells (40,000 cells/well) were co-cultured with SMAD-binding element (SBE)-HEK293 cells (40,000 cells/well; BPS Bioscience San Diego, CA) in 96-well assay plates (Sigma-Aldrich) using DMEM supplemented with 0.5% FBS. After 18 hours, the SBE activity was analyzed using the One-Step Luciferase Assay System (BPS Bioscience) on a Glomax Multi Detection System (Promega) following the manufacturer’s instructions.

*Analysis of cell proliferation in vitro*

Cell proliferation was analyzed using the xCelligence real-time cell analyzer system from Roche (Roche Applied System, Penzberg, Germany) as previously described (1). In some experiments, proliferation was measured as follows: 50,000 cell/well of non-transduced (NT) and GARP-overexpressing (GARP^++^) G292, SAOS-2, and RD-ES cells were plated in 12-well plates. When the cell cultures reached 80-90% of confluence, cells were harvested, counted, and re-plated at the same concentration. In some cases, 5 hours after cell plating, cells were treated with 10 μM of the ALK4/ALK5/ALK7 inhibitor, SB431542 (Sigma-Aldrich, St. Louis, MO), or 2.5 µg/ml of anti-TGF-β1/2/3 Ab (11D1, R&D Systems, Minneapolis, MN).

*Cell viability in response to etoposide and doxorubicin*

Cell viability was measured using the CellTiter-Blue reagent (Promega, Madison, WI) following the manufacturer´s instructions. Briefly, 10,000 cells/well of NT, GARP^++^, and EGFP-overexpressing (EGFP^++^) G292, SAOS-2, and RD-ES cells were plated in 96-well plates. In some experiments, 10 μM of SB431542 or 2.5 µg/ml of anti-TGF-β1/2/3 antibody were added to the cells five hours after plating. After 24 hours, etoposide (Sigma-Aldrich) or doxorubicin (Enzo Life Sciences, Farmingdale, NY) were added (2.5, 5, 10, and 20 µM for RD-ES and SAOS-2 cells and 10, 20, 40, and 80 µM for G292 cells). CellTiter-Blue was added 24 and 48 hours after etoposide/doxorubicin addition to the RD-ES and G292/SAOS-2 cultures, respectively. Fluorescence was measured four hours later at 560 nm on a Glomax Multi Detection System (Promega) following the manufacturer’s instructions. In addition, apoptosis was measured as described in supplementary materials and methods.

*7AAD/Annexin V Staining*

Apoptosis was analyzed by flow cytometry on day 5 after GARP-silencing using the 7AAD/PE Annexin V Apoptosis Kit I (BD Biosciences, San Diego, USA) according to the manufacturer´s instructions. In order to study the effect of the overexpression of GARP on etoposide-induced apoptosis, 50,000 cells/well of NT and GARP^++^ G292, SAOS-2 and RD-ES tumor cells were plated in 12-well plates. 24 hours later, cells were treated with different concentrations of etoposide (Sigma-Aldrich) for 24 hours and stained with Annexin V as explained above.

*Analysis of H2AX phosphorylation*

For the quantification of double-strand DNA breaks (DSBs), phosphorylated H2AX (γ-H2AX) was measured by flow cytometry using the FlowCellect® Histone H2AX Phosphorylation Assay Kit (Millipore, Burlington, MA). To induce DSBs, NT and GARP^++^ SAOS-2, and RD-ES tumour cells were seeded in 12-well plates (50,000 cells/well), with or without SB431542 (10 μM) and the next day exposed to a single radiation dose of 4 Gy. After 2 hours, cells were harvested and stained for γ-H2AX according to the manufacturer´s instructions. Cells were acquired on a FACS Canto II (BD Bioscience) and analyzed by the FACS Diva software (BD Bioscience) or on an ImageStream X Mark II Imaging flow cytometer (Millipore) and analyzed using the IDEAS software (Spot Wizard).

*Immunohistochemical staining and quantification of Ki67 and phospho-SMAD3 in G292 tumors*

The procedure was performed automatically using Ventana BenchMark XT and the ultraView Universal DAB Detection Kit (Ventana Medical Systems), in line with the producer's manual. Heat-induced epitope retrieval (HIER) was performed with cell conditioning 1 (CC1, pH 8.3). Ki-67 was incubated at a 1:75 dilution after 60 minutes of HIER treatment of the tissue. SMAD3 was incubated at a 1:300 dilution after 32 minutes of HIER treatment. For each sample, a Hematoxilin-Eosin (HE) stain of an adjacent slide was also performed. Virtual slides were obtained by means of a Nanozoomer 2.ORS scanner and were viewed and analyzed both using its NDP.Viewer or the Aperio Imagescope software. Tissue areas were measured, distinguishing between the total area and the area of necrotic tissue, if any. Regarding the analysis of the marker’s expression, since cell line induced tumors are highly homogeneous, three representative areas of the same size were selected and analyzed using the Pixel Count V9.1 algorithm of the Aperio Imagescope software. Values for three different positive pixel intensities (NWP=weak positive, NP= positive and NSP=strong), along with values for negative (Nn) and total pixels (NTotal) were obtained for each area. Mean values and standard deviations of all variables from the three analyzed areas were obtained per sample. The percentage of positive pixels was also calculated, considering the sum of positive and strongly positive pixels (NP+NSP).

*Tissue microarray construction*

Formaldehyde fixed and paraffin embedded tissues were cut and stained with H&E, with the aim of selecting three representative tumor regions from each case. To avoid cross-contamination, two punches of 2 mm diameter were taken first, using a new, sterile punch (Kai Europe GmbH, Solingen, Germany) for every tissue block, and stored in Eppendorf tubes at room temperature to be used for DNA extraction. Then 1 mm cores were extracted to make tissue microarrays (TMA) containing three tissue cores from each of the 89 sarcoma samples. A manual tissue arrayer (Beecher Instruments, Sun Prairie, WI, USA) was used. After five minutes at 60ºC, the TMA blocks were cut in 4 μm-thick sections for immunohistochemical techniques. Normal epithelial tissue, present in each TMA, was considered as a reference.

*GARP/LRRC32 immunohistochemistry in human sarcoma TMA*

Formalin-fixed, paraffin-embedded tissues were cut into 3-μm sections and dried on Flex IHC microscope slides (Dako, Glostrup, Denmark). Sections were deparaffinized with standard xylene and hydrated through graded alcohols into water. Antigen retrieval was performed using Envision Flex Target Retrieval solution, low pH (Dako, Glostrup, Denmark). Stainings were performed at room temperature on an automatic staining workstation (Dako Autostainer Plus) with an anti-LRRC32/GARP antibody (Abcam Cat#: ab231214) at 1:300 dilution using the Dako EnVision Flex + Visualization System (Dako Autostainer, Denmark). Counterstaining with hematoxylin was the final step. Immunostaining was scored blinded to clinical data by two independent observers, using a semiquantitative scoring system based on both the percentage of stained cells (1: 0%; 2: <10%; 3: 10-50%; and 4: >50%) and the staining intensity (0: no expression; 1: low intensity; and 2: high intensity). Each sample received a scoring value resulting from the multiplication of both scores, and the median value in the resulting distribution was used to discriminate low and high expressing samples.

REFERENCES SUPPLEMENTARY MATERIALS AND METHODS

1. Carrillo-Galvez AB, Cobo M, Cuevas-Ocana S, Gutierrez-Guerrero A, Sanchez-Gilabert A, Bongarzone P, et al. Mesenchymal stromal cells express GARP/LRRC32 on their surface: effects on their biology and immunomodulatory capacity. Stem Cells. 2015 Jan;33(1):183-95.
