# Supplementary material for: GARP promotes the proliferation and therapeutic resistance of bone sarcoma cancer cells through the activation of TGF-β
Source: Cell Death Dis. 2020 Nov 17;11(11):985. doi: 10.1038/s41419-020-03197-z (PMC7673987; doi:10.1038/s41419-020-03197-z)
Supplement: Supplementary file 2 — Supplementary Figure Legends [file 41419_2020_3197_MOESM2_ESM.docx]

**SUPPLEMENTARY FIGURE LEGENDS**

**Figure S1. Silencing of GARP in bone sarcoma cell lines.** (A) GARP expression on G292, BM-MSCs, T1-73, SAOS-2 and RD-ES cells were measured by FACS. Representative dot plots show GARP expression as % GARP-positive cells in relation to isotype control stainings (horizontal lines). G292, T1-73 and SAOS-2 cell lines were transduced with LV-CTRL, LV-GARP^KO1^ and LV-GARP^KO2^ as described in the materials and methods. Four days later, GARP expression was measured by FACS and compared to non-transduced (NT) cells as described in the supplementary materials and methods. Data show (B) the % of GARP^+^ cells in relation to isotype control or (C) the geometric mean fluorescent intensity (GeoMean) of isotype (grey bars) and GARP (black, white, dark and light blue bars) stainings. Data are represented as mean(SEM) of two (T1-73) or three (G292, SAOS-2) independent experiments.

**Figure S2. Representative dot plots showing GARP expression on sarcoma cell lines, before and after GARP silencing.** NT, LV-CTRL, GARP^KO1^ and GARP^KO2^ G292, T1-73, and SAOS-2 cells were stained with a rat IgG2a kappa-eFluor660 isotype control (NT; left dot plots) and an anti-human GARP-eFluor660 Ab (NT, LV-CTRL, GARP^KO1^ and GARP^KO2^). Representative dot plots are shown from one out of two (T1-73) or three (G292, SAOS-2) independent experiments.

**Figure S3. Measurement of active TGF-β in supernatants from non-transduced (NT) and GARP-overexpressing (GARP^++^) sarcoma cells.** Supernatants from NT and GARP^++^ G292, SAOS-2, and RD-ES cells and recombinant TGF-β1 (1 ng/ml) were added to SMAD-binding element (SBE)-HEK293 cells and the luciferase activity were read after 18 hours as described in the materials and methods. Data are shown as mean(SD) of at least three independent experiments.

**Figure S4. GARP-overexpressing G292, SAOS-2 and RD-ES cells are more resistant to etoposide induced apoptosis in comparison to NT cells.** NT and GARP^++^ G292, SAOS-2 and RD-ES cells were exposed to different concentrations of etoposide (Etop) for 24 hours and stained for Annexin V/7AAD, as described in supplementary materials and methods, and analyzed by flow cytometry. (A) Gating strategy for the analysis of apoptotic cells by flow cytometry. The dot plots show the Annexin V and 7AAD staining of NT and GARP^++^ G292 cells based on all acquired events. Apoptotic cells were defined as Annexin V^+^/7AAD^-/+^ cells, shown in gate “P2”. (B) Summary of apoptosis induction in G292, SAOS-2 and RD-ES cells. Data are shown as mean (SD) of at least three individual experiments. *=P<0.05, **=P<0.01.

**Figure S5. GARP silencing in the primary tumor-derived cell line OST-4 and GARP control stainings of human tumors.** (A) Representative histograms showing the geometric mean fluorescent intensity of isotype and GARP staining of non-transduced (NT), LC-CTRL and LV-GARP^KO2^ transduced OST-4 cells. (B, C) Immunohistochemical controls of GARP stainings. (B) GARP staining in a sample of urothelial papillary carcinoma, showing areas of strong (P) and low/negative (N) GARP staining. (C) GARP staining in a dermatofibrosarcoma sample showing positive staining in endothelial vascular cells (internal positive control, red arrows) but not in tumor cells.

**Table S1.** Distribution of sarcoma cases (N=89) according to their GARP expression level across categories of the indicated patient characteristics and tumor clinicopathological parameters. P values are shown.

**Table S2.** Univariate and multivariate Cox analysis of GARP expression and clinicopathological parameters, including tumor necreosis, tumor grade tumor count and tumor type.

**Table S3.** Patients (N=11) with available clinical history suffering from various sarcoma subtypes were treated with different first line chemotherapy treatments (ChTP1). CR: Complete Response, PR: Partial Response, SD: Stable Disease and DP: Disease Progression.

**Table S4.** Dichotomous analysis of patients with high and low GARP expression and their responses to chemotherapy (RespChTP1). CR: Complete Response, PR: Partial Response, SD: Stable Disease and DP: Disease Progression.
