# Supplementary figures and images for: GARP promotes the proliferation and therapeutic resistance of bone sarcoma cancer cells through the activation of TGF-β
Source: Cell Death Dis. 2020 Nov 17;11(11):985. doi: 10.1038/s41419-020-03197-z (PMC7673987; doi:10.1038/s41419-020-03197-z)

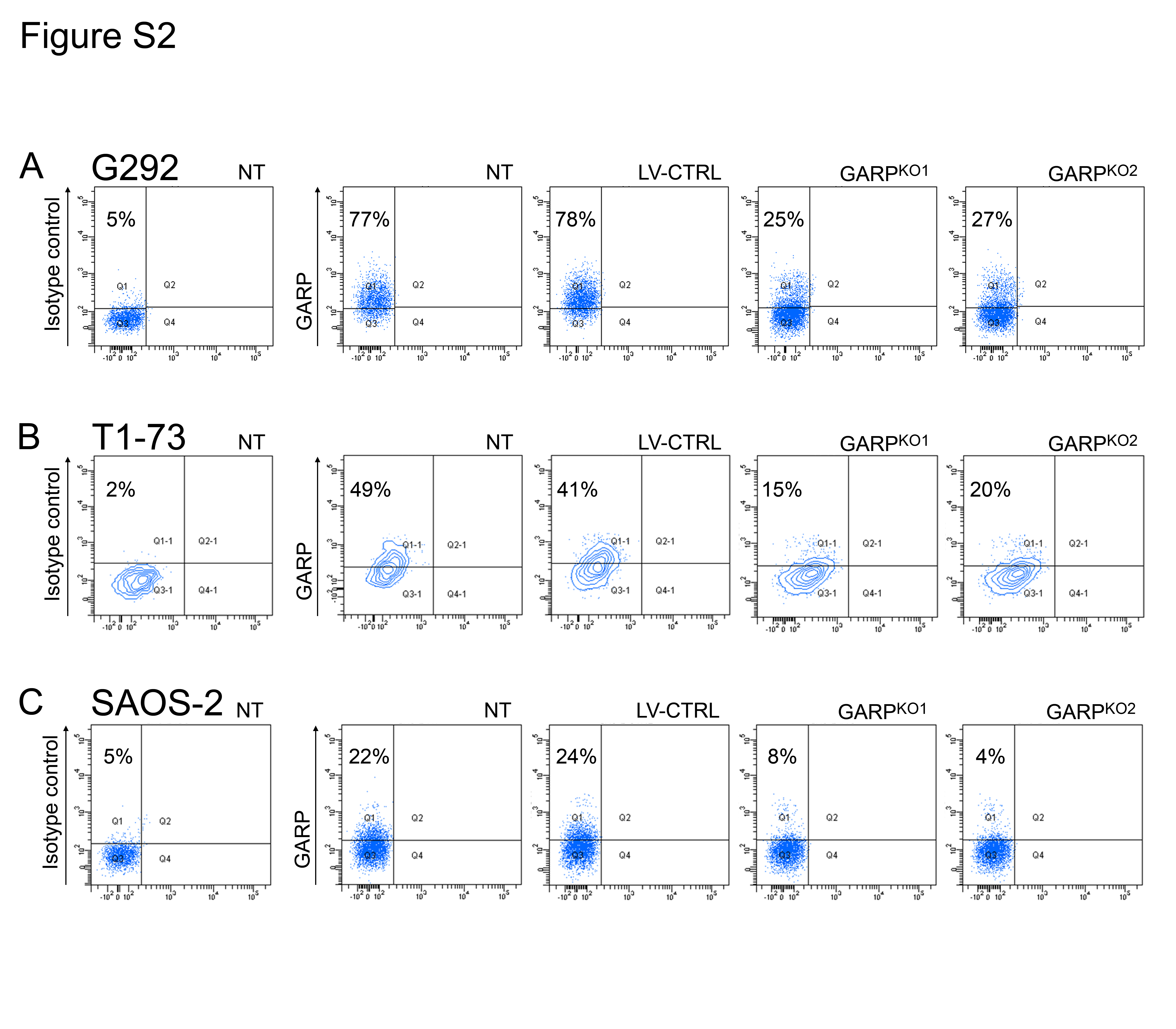

Supplement: Supplementary file 4 — Figure S2 [file 41419_2020_3197_MOESM4_ESM.tif]

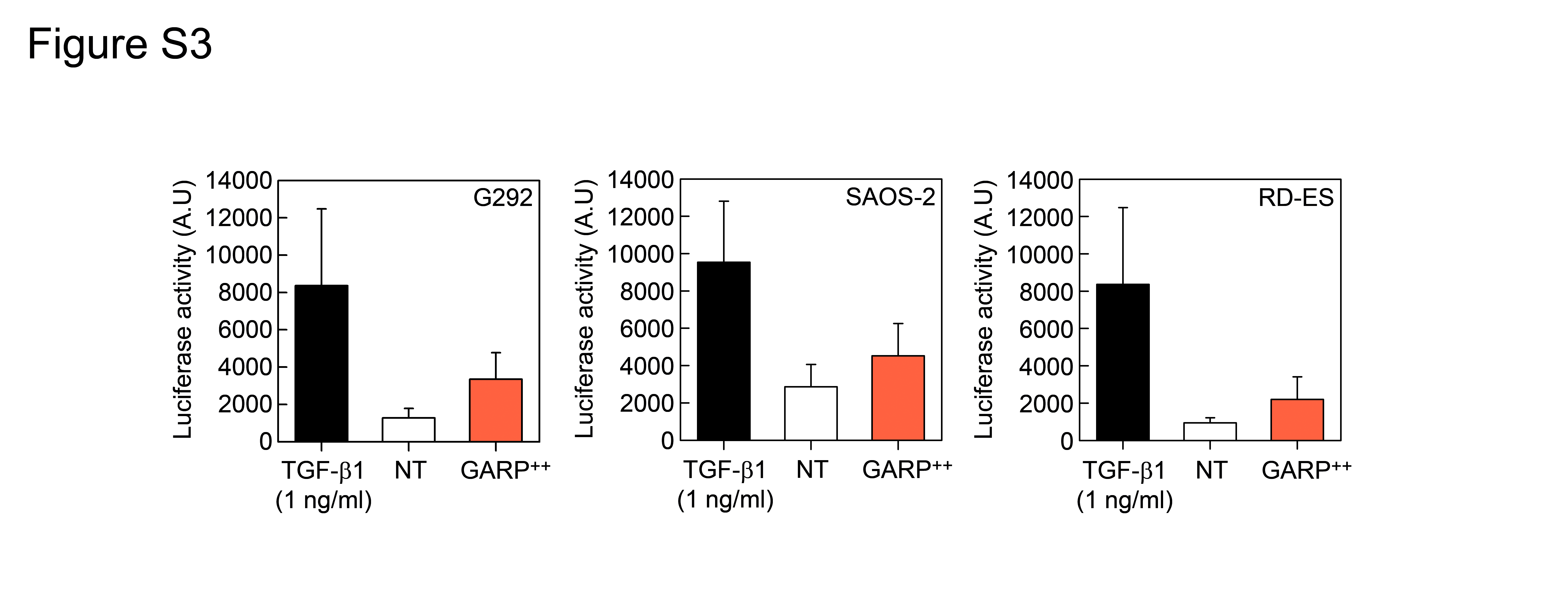

Supplement: Supplementary file 5 — Figure S3 [file 41419_2020_3197_MOESM5_ESM.tif]

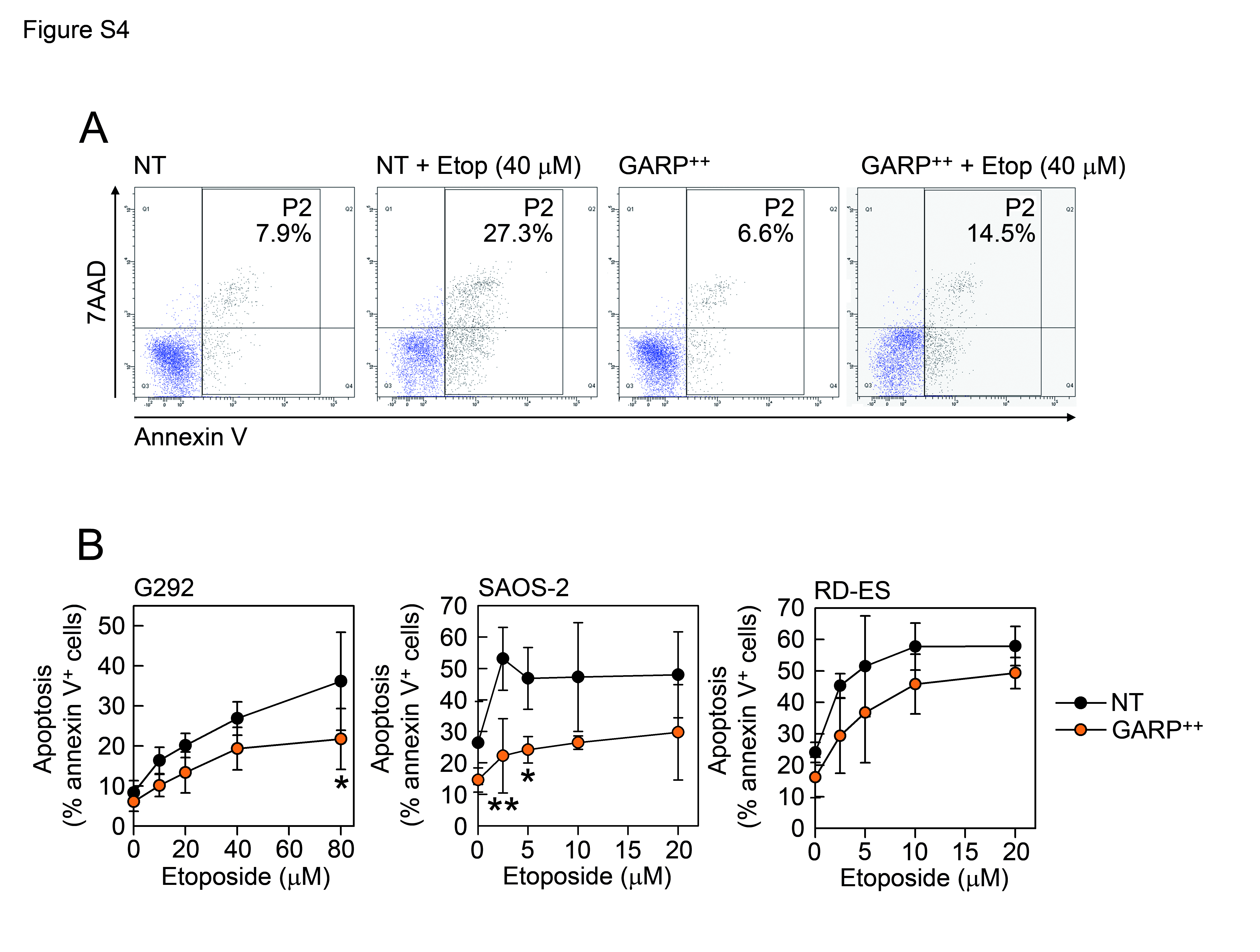

Supplement: Supplementary file 6 — Figure S4 [file 41419_2020_3197_MOESM6_ESM.tif]

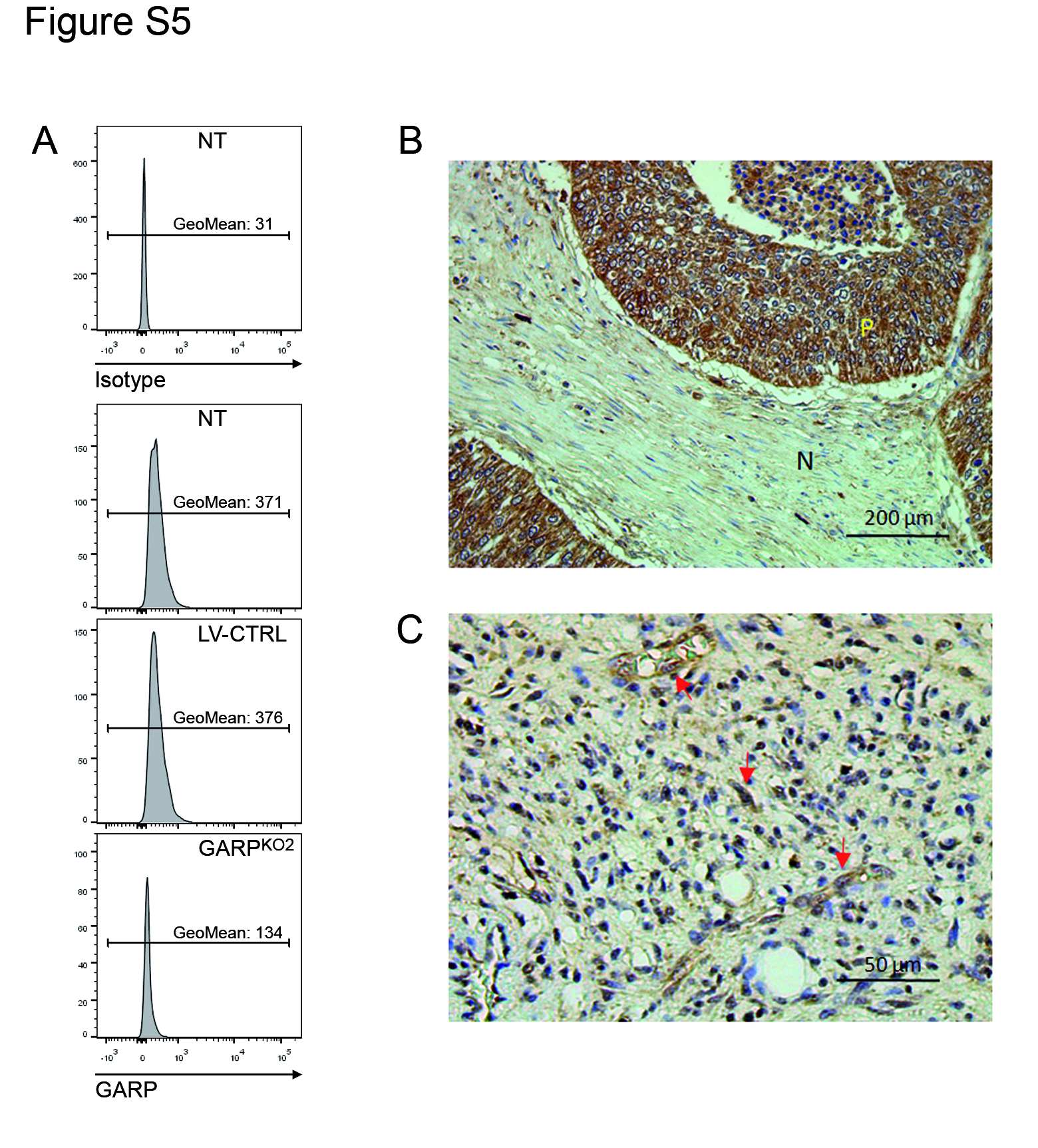

Supplement: Supplementary file 7 — Figure S5 [file 41419_2020_3197_MOESM7_ESM.tif]
