# Supplementary material for: GARP promotes the proliferation and therapeutic resistance of bone sarcoma cancer cells through the activation of TGF-β
Source: Cell Death Dis. 2020 Nov 17;11(11):985. doi: 10.1038/s41419-020-03197-z (PMC7673987; doi:10.1038/s41419-020-03197-z)
Supplement: Supplementary file 8 — Supplementary Table Legends [file 41419_2020_3197_MOESM8_ESM.docx]

**SUPPLEMENTARY TABLE LEGENDS**

**Table S1.** Distribution of sarcoma cases (N=89) according to their GARP expression level across categories of the indicated patient characteristics and tumor clinicopathological parameters. P values are shown.

**Table S2.** Univariate and multivariate Cox analysis.

**Table S3**. Patients (N=11) with available clinical history suffering from various sarcoma subtypes were treated with different first line chemotherapy treatments (ChTP1). CR: Complete Response, PR: Partial Response, SD: Stable Disease and DP: Disease Progression, PNET: Peripheral neuroectodermal tumour, GIST: Gastrointestinal stromal tumour.

**Table S4.** Dichotomous analysis of patients with high and low GARP expression and their responses to chemotherapy (RespChTP1). CR: Complete Response, PR: Partial Response, SD: Stable Disease and DP: Disease Progression.
