# Supplementary material for: GARP promotes the proliferation and therapeutic resistance of bone sarcoma cancer cells through the activation of TGF-β
Source: Cell Death Dis. 2020 Nov 17;11(11):985. doi: 10.1038/s41419-020-03197-z (PMC7673987; doi:10.1038/s41419-020-03197-z)
Supplement: Supplementary file 9 — Supplementary Table 1 [file 41419_2020_3197_MOESM9_ESM.docx]

**Table S1.**

|  | **Low GARP (%)** | **High GARP (%)** | **Total** | **P (Chi-Square)** |
| --- | --- | --- | --- | --- |
| **Diagnosis** |  |  |  |  |
| Osteosarcoma | 0 (0) | 8 (100) | 8 | 0.0001 |
| Ewing Sarcoma | 5 (83) | 1 (17) | 6 |  |
| Myxoid Liposarcoma | 10 (71) | 4 (29) | 14 |  |
| Liposarcoma | 6 (75) | 2 (25) | 8 |  |
| Chondrosarcoma | 3 (27) | 8 (73) | 11 |  |
| Synovial Sarcoma | 7 (78) | 2 (22) | 9 |  |
| Pleomorphic Sarcoma | 0 (0) | 10 (100) | 10 |  |
| Enchondroma | 5 (56) | 4 (44) | 9 |  |
| Dermatofibrosarcoma | 9 (100) | 0 (0) | 9 |  |
| GIST | 1 (20) | 4 (80) | 5 |  |
| Total | 46 | 43 | 89 |  |
| **Tumor size** |  |  |  |  |
| < 9,65 cm | 20 (55) | 16 (45) | 36 | 0.407 |
| >9,65 cm | 16 (46) | 19 (54) | 35 |  |
| Total | 36 | 35 | 71 |  |
| Missing | 10 | 8 | 18 |  |
| **Grade** |  |  |  |  |
| 1 | 10 (63) | 6 (37) | 16 | 0.15 |
| 2 | 9 (45) | 11 (55) | 20 |  |
| 3 | 6 (30) | 14 (70) | 20 |  |
| Total | 25 | 31 | 56 |  |
| Missing | 21 | 12 | 33 |  |
| **Differentiation degree** |  |  |  |  |
| Well | 7 (70) | 3 (30) | 10 | 0.217 |
| Moderate | 2 (40) | 3 (60) | 5 |  |
| Poor | 12 (39) | 19 (61) | 31 |  |
| Total | 21 | 25 | 46 |  |
| Missing | 18 | 25 | 43 |  |
| **Mitotic count** |  |  |  |  |
| 0 | 0 (0) | 2 (100) | 2 | 0.068 |
| 1 | 20 (49) | 14 (51) | 34 |  |
| 2 | 3 (25) | 9 (75) | 12 |  |
| 3 | 3 (30) | 7 (70) | 10 |  |
| Total | 26 | 32 | 58 |  |
| Missing | 20 | 11 | 31 |  |
| **Tumor necrosis** |  |  |  |  |
| No | 16 (47) | 18 (53) | 34 | 0.342 |
| <50% | 7 (50) | 7 (50) | 14 |  |
| >50% | 1 (17) | 5 (83) | 6 |  |
| Total | 24 | 30 | 54 |  |
| Missing | 22 | 13 | 35 |  |
| **Vascular invasion** |  |  |  |  |
| No | 22 (44) | 28 (56) | 50 | 0.954 |
| Yes | 3 (43) | 4 (57) | 7 |  |
| Total | 25 | 32 | 57 |  |
| Missing | 21 | 11 | 32 |  |
| **Lymphatic invasion** |  |  |  |  |
| No | 24 (45) | 29 (55) | 53 | 0.431 |
| Yes | 1 (25) | 3(75) | 4 |  |
| Total | 25 | 32 | 57 |  |
| Missing | 21 | 11 | 32 |  |
| **Neural invasion** |  |  |  |  |
| No | 21 (43) | 28 (57) | 49 | 0.842 |
| Yes | 1 (50) | 1 (50) | 2 |  |
| Total | 22 | 29 | 51 |  |
| Missing | 24 | 14 | 38 |  |
