# Supplementary material for: GARP promotes the proliferation and therapeutic resistance of bone sarcoma cancer cells through the activation of TGF-β
Source: Cell Death Dis. 2020 Nov 17;11(11):985. doi: 10.1038/s41419-020-03197-z (PMC7673987; doi:10.1038/s41419-020-03197-z)
Supplement: Supplementary file 10 — Supplementary Table 2 [file 41419_2020_3197_MOESM10_ESM.docx]

**Table S2.**

| **Variable** | **P value univariate**  **analysis** | **P value multivariate**  **analysis** |
| --- | --- | --- |
| Tumor necrosis | 0.041 | 0.024 |
| GARP expression | 0.000 | 0.056 |
| Tumor grade | 0.012 | 0.42 |
| Mitotic count | 0.055 | 0.693 |
| Tumor type | 0.263 | 0.955 |
