# Supplementary material for: GARP promotes the proliferation and therapeutic resistance of bone sarcoma cancer cells through the activation of TGF-β
Source: Cell Death Dis. 2020 Nov 17;11(11):985. doi: 10.1038/s41419-020-03197-z (PMC7673987; doi:10.1038/s41419-020-03197-z)
Supplement: Supplementary file 11 — Supplementary Table 3 [file 41419_2020_3197_MOESM11_ESM.docx]

**Table S3**.

| **Sarcoma type** | **ChTP1** | **Response** |
| --- | --- | --- |
| Ewing sarcoma | vincristine-ifosfamide-doxorubicin-etoposide | CR |
| biphasic synovial sarcoma | ifosfamide-doxorubicin | SD |
| pleomorphic sarcoma | ifosfamide- liposomal doxorubicin | PR |
| myxoid liposarcoma | ifosfamide-doxorubicin (x7) and ifosfamide monotherapy (x4) | PR |
| monophasic synovial sarcoma | ifosfamide-doxorubicin | CR |
| PNET | ifosfamide-etoposide-vincristine-doxorubicin- cyclophosphamide | DP |
| GIST | Imatinib | PR |
| GIST | Imatinib | PR |
| GIST | Imatinib | PR |
| GIST | Imatinib | PR |
| GIST | Imatinib | PR |
