# Supplementary material for: GARP promotes the proliferation and therapeutic resistance of bone sarcoma cancer cells through the activation of TGF-β
Source: Cell Death Dis. 2020 Nov 17;11(11):985. doi: 10.1038/s41419-020-03197-z (PMC7673987; doi:10.1038/s41419-020-03197-z)
Supplement: Supplementary file 12 — Supplementary Table 4 [file 41419_2020_3197_MOESM12_ESM.docx]

**Table S4.**

| P=0.039 | RespChTP1 |  |  |
| --- | --- | --- | --- |
| **GARP** | **CR** | **PR+SD+DP** | Total |
| Low | 2 | 2 | 4 |
| High | 0 | 7 | 7 |
| **Total** | 2 | 9 | 11 |
